# Electrochemical Oxidation/Disinfection of Urine Wastewaters with Different Anode Materials

**DOI:** 10.3390/ma12081254

**Published:** 2019-04-16

**Authors:** Sondos Dbira, Nasr Bensalah, Mohammad I. Ahmad, Ahmed Bedoui

**Affiliations:** 1 Department of Chemistry, Faculty of Sciences of Gabes, University of Gabes, 6072 Gabes, Tunisia; sondos.dbira@gmail.com (S.D.); ahmed.bedouifsg@yahoo.fr (A.B.); 2 Department of Chemistry and Earth Sciences, College of Arts and Science, Qatar University, P.O. Box 2713, Doha, Qatar; 3 Central Laboratories Unit, Qatar University, P.O. Box 2713, Doha, Qatar; mohammad.ibrahim@qu.edu.qa

**Keywords:** Urine wastewaters, microorganisms, anode material, deactivation, mineralization

## Abstract

In the present work, electrochemical technology was used simultaneously for the deactivation of microorganisms and the destruction of micro-pollutants contained in synthetic urine wastewaters. Microorganisms (*E. coli*) were added to synthetic urine wastewaters to mimic secondary treated sewage wastewaters. Different anode materials were employed including boron-doped diamond (BDD), dimensionally stable anode (DSA: IrO_2_ and RuO_2_) and platinum (Pt). The results showed that for the different anode materials, a complete deactivation of *E. coli* microorganisms at low applied electric charge (1.34 Ah dm^−3^) was obtained. The complete deactivation of microorganisms in wastewater seems to be directly related to active chlorine and oxygen species electrochemically produced at the surface of the anode material. Complete depletion of COD and TOC can be attained during electrolyses with BDD anode after the consumption of specific electric charges of 4.0 and 8.0 Ah dm^−3^, respectively. Higher specific electric charges (>25 Ah dm^−3^) were consumed to removal completely COD and about 75% of TOC during electrolyses with DSA anodes (IrO_2_ and RuO_2_). However, the electrolysis using Pt anode can partially remove and even after the consumption of high specific electric charges (>40 Ah dm^−3^) COD and TOC did not exceed 50 and 25%, respectively. Active chlorine species including hypochlorite ions and chloramines formed during electrolysis contribute not only to deactivate microorganisms but also to degrade organics compounds. High conversion yields of organic nitrogen into nitrates and ammonium were achieved during electrolysis BDD and DSA anodes. The results have confirmed that BDD anode is more efficient than with IrO_2_, RuO_2_ and Pt electrodes in terms of COD and TOC removals. However, higher amounts of perchlorates were measured at the end of the electrolysis using BDD anode.

## 1. Introduction

Human urine represents a small volume of domestic wastewater but it is one of the main sources of water pollution. Urine is characterized by a very complex chemical composition including both ionic and molecular compounds. Several researchers have investigated the composition of human urine [1,2]. The organic fraction of urine is composed principally of urea, uric acid and creatinine. It contains also most of the essential nutrients nitrogen, phosphorus and potassium and very low metals content [1,3,4]. In addition, certain hormones and medicinal residues are excreted with the urine, which find themselves in the sewers and it has been considered as an emerging environmental problem [5]. On the other hand, Heinonen-Tanski et al. [2] reported that a possible transmission of pathogens could result from the reuse of inadequately treated human and animal wastes in irrigation or as fertilizers in agriculture. Consequently untreated urine wastewater causes major damage to the environment and to human health. For this reason, it is critical to develop more efficient and cost-effective method of treatment capable to treat the wastewater before its reuse in irrigation or discharge into water bodies.

In recent years, electrochemical technologies become good candidates to replace conventional methods for treating wastewaters containing toxic and refractory organic pollutants [6,7,8,9]. The effectiveness of electrochemical methods is mainly related to the nature of the anode materials [10,11]. It is important that the electrode material should be appropriately chosen to ensure highly efficient electrochemical treatment. One of the most interesting electrochemical wastewater treatment technologies is electrochemical oxidation (EO) [12,13,14,15,16]. Electrochemical Oxidation (EO) is the most performant electrochemical method for mineralizing persistent and bio-resistant organic pollutants in water and wastewater including phenol derivatives and others [8,17,18,19,20,21]. In this context, we have recently investigated the electrochemical oxidation of synthetic urine using BDD and DSA anodes, confirming that EO is a suitable method to mineralize organic content from synthetic urine and it can also efficiently convert organic nitrogen into nitrate and ammonium ions [22,23,24,25]. However, the main drawback of this technology is that it cannot prevent the oxidation of hypochlorite ions into hazardous disinfection by-products: chlorates and perchlorates. It is then necessary to search another method to integrate with electrochemical oxidation to consume hypochlorite ions and then avoid its oxidation into chlorates and perchlorates during electrochemical treatment. Electrochemical disinfection has been reported to be an effective method in killing many pathogenic microorganisms in wastewaters thanks to the high production of oxidants achieved [26,27,28,29,30,31,32]. It is essential also to control operational conditions to prevent the formation of hazardous disinfection by-products [22,23,33,34,35,36]. This process involves principally the elimination of faecal coli forms present in water by the contribution of direct and indirect routes in the disinfection mechanisms: 1) the adsorbed *Escherichia coli* (*E. coli*) on the surface of the anode are killed/deactivated by direct electrochemical oxidation [32,37,38] and 2) the strong oxidants formed during electrolysis deactivate and destruct *E. coli* cells in solution [39,40,41]. The effectiveness of the electrodisinfection varies with the concentration of the pollutants and microorganisms, the electric current applied and the nature of anode materials. Boron doped diamond (BDD) and dimensionally stable anodes (DSA) are commonly used as anode materials during electrochemical disinfection [29,42,43,44,45].

Boron Doped Diamond (BDD) electrode has unique characteristics such as chemical and electrochemical stability and large electro-activity window with high over potential for O_2_ evolution. These enables large production of hydroxyl radicals by water discharge on BDD surface (Equation (1)) [12,46,47,48,49]. 

BDD + H_2_O → BDD (HO^•^) + H^+^ + 1e^−^ (1)

Dimensionally stable anodes (DSAs) are largely utilized for electrochemical treatment of water and wastewater in industrial plants [50,51,52,53,54]. DSA anodes present high performance in the production of hypochlorite ions for saline waters [55,56,57]. Recently, both BDD and DSAs anodes achieved complete mineralization of numerous organic pollutants [53,55,56].

The goal of this work was to investigate the electrochemical oxidation/disinfection of synthetic urine wastewaters in laboratory simulating sewage wastewaters for larger applications. Cotillas et al. [26,27,28,29,30,31,32] have recently investigated the electrochemical disinfection of urine wastewaters using conductive diamond anodes. Although excellent electrochemical performance was obtained with diamond anodes, large amounts of chlorates and perchlorates were measured at the end of the treatment. The present work aims to understand the role of the anode material on the performance of the combined electrooxidation/electrodisinfection process and the formation of disinfection by-products during the electrochemical treatment. The performance of the electrochemical treatment was evaluated by monitoring the changes in total organic carbon (TOC), chemical oxygen demand (COD), pH and conductivity. Furthermore, high performance liquid chromatography (HPLC) analysis was used to detect the formation of intermediates and the degradation of initial products. The identification and quantification of the inorganic ions formed during galvanostatic electrolyses were performed by ion chromatography. The efficiencies of electrolysis with different anode materials were compared in terms of deactivation of microorganism (*E. coli*), COD and TOC removals and formation of chlorate and perchlorate ions. 

## 2. Materials and Methods 

### 2.1. Chemicals

The synthetic urine solutions were prepared by dissolving urea 0.996 g/L (CH_4_N_2_O), 0.015 g/L uric acid (C_5_H_4_N_4_O_3_) and 0.0498 g/L creatinine (C_4_H_7_N_3_O). Salts were also dissolved to obtain 0.3 g/L chloride (Cl^−^), 0.3 g/L potassium (K^+^), 0.0075 g/L phosphate (PO_4_^3−^), 0.051 g/L sulphate (SO_4_^2−^), 0.0075 g/L calcium (Ca^2+^), 0.051 g/L magnesium (Mg^2+^), 0.04998 g/L sodium (Na^+^), 0.0075 g/L ammonium (NH_4_^+^) and 0.04998 g/L carbonates (CO_3_^2−^). Urea (CH_4_N_2_O), uric acid (C_5_H_4_N_4_O_3_) and creatinine (C_4_H_7_N_3_O) were analytical grade (>99.0% purity), purchased from Sigma-Aldrich. All additional chemicals were obtained from Fluka. Deionized water obtained from a Millipore Milli-Q system (with resistivity >18 MΩ cm^−1^ at 25 °C) was used to prepare all the aqueous solutions.

### 2.2. Analytical Methods

The measurements of total organic carbon (TOC) and total nitrogen (TN) were performed using a Multi N/C 3100 Analytik Jena TOC analyser. Conductivity and pH were monitored using a GLP 31 Crison conductimeter and, an InoLab WTW pH-meter, respectively. Chlorine species (Cl^−^, ClO_3_^−^ and ClO_4_^−^) were analysed by ion chromatography using a Shimadzu LC-20A equipped with Shodex IC I-524A column. 2.5 mM phthalic acid (pH 4.0) was used as mobile phase at 1.0 mL min^−1^ flow rate. Nitrate, nitrite, NH_4_^+^, sulphate and phosphate were quantified by the same ion chromatograph (column: Shodex IC I-524A for anions and Shodex IC YK-421 for cations), however, the mobile phase was an aqueous solution containing 5.0 mM tartaric, 1.0 mM dipicolinic acid and 24.3 mM boric acid (flow rate of 1.0 mL min^−1^). Hypochlorite was titrated with 0.001 M As_2_O_3_ in 2 M NaOH. High performance liquid chromatography, HPLC (Agilent 1100 series) equipment was employed to identify and quantify urine organic content before and during electrochemical treatment. 20 μL of each sample was injection in a Phenomenex Gemini 5 μm C18 column kept at 25 °C. The UV detector was set at 291 nm. The mobile phase consists of mixture of solvent A (25 mM formic acid aqueous solution) and solvent B (acetonitrile). The composition of mobile phase in solvent B was linearly increased starting from 10% to reach 100% within 40 min. Before HPLC analysis, all the samples withdrawn during the electrochemical treatment underwent filtration through 0.20 μm membranes. The analysis of chloramines was performed using DPD colorimetric method (4500-Cl G) by UV-2400PC Shimadzu spectrophotometer [58]. The faecal coliforms from wastewaters were estimated using the most probable number (MPN) technique [59]. Microorganism counts were carried out by the multiple-tube-fermentation technique (24 h of incubation at 44 °C) using 5 tubes at each dilution (1:10, 1:100 and 1:1000). Chemical oxygen demand (COD) was analysed by digestion of a mixture of 2 mL water sample with dichromate acidic solution as the oxidizing agent (Merck-Spectroquant^®^) during 2 h. COD was determined by UV-visible spectrophotometry using a UV-1603 Shimadzu spectrophotometer in 1 cm quartz cells.

### 2.3. Voltammetry Experiments

Linear sweep voltammetry (LSV) was carried out in a typical three-electrode system on a potentiostat/galvanostat (PGSTAT-30 Autolab) connected to a computer as an electrochemical workstation. Pt, IrO_2_, RuO_2_ and BDD electrodes of surface area 1 cm^2^ (De Nora Germany) of surface area 1 cm^2^ (Adamant Technologies) were used as the working electrode; saturated calomel electrode (SCE) was used as reference electrode and a graphite rod was used as counter electrode. All measurements were performed at room temperature 20 mL electrochemical cells. LSV was repeated until reproducible voltammograms were obtained. The voltammogram were recorded up to the decomposition potential of water and/or the supporting electrolyte. 

### 2.4. Electrochemical Set Up

A single compartment electrochemical flow cell working under a batch-operation mode was used as the electrochemical reactor for all the experiments. The different anode materials (BDD, DSA and Pt) were used in this work in parallel with a stainless steel (AISI 304) as cathode material an inter-electrode gap of 9 mm. BDD films were provided by Adamant Technologies (Neuchatel, Switzerland) and synthesized by the hot filament chemical vapor deposition technique (HF CVD) on single-crystal p-type Si (100) wafers (0.1 Ω cm Siltronix), (Si substrate; 2 mm thickness; 100 mΩ cm resistivity; 500 ppm boron concentration; 2.62 μm CDE-film thickness; 115 CDE-film Raman sp^3^/sp^2^). The DSA anodes studied were DSA-Cl_2_: ruthenium oxide on titanium (Ti/RuO_2_), DSA-O_2_: iridium oxide on titanium (Ti/IrO_2_) and Ti–Pt, all of them supplied by DeNora, Italy. 100 mm-diameter circular disks (78 cm^2^ geometric area) electrodes with 9 mm inter-electrode distance were used in all the electrochemical experiments. 

### 2.5. Experimental Procedures

The electrochemical treatment of urine wastewaters were carried out under galvanostatic mode. 600 mL urine aqueous solution was treated in each electrochemical experiment. A current density of 15 mA cm^−2^ was applied between the two electrodes. In all experiments, the initial pH of the solution was fixed to 8.0 (±0.5). A stable cell voltage was measured during electrolysis indicating no appreciable deterioration or passivation of electrode took place. In order to eliminate any impurity adsorbed on the surface, the electrodes were pre-treated by polarization in an aqueous solution of Na_2_SO_4_ (0.035 M) during 10 min at 15 mA cm^−2^. A centrifugal pump with a constant flow rate was used to circulate urine aqueous solution initially stored in a glass tank through the electrolytic cell. The temperature was maintained at 25 °C by water circulation from a controlled thermostatic bath (Digiterm 100, JP Selecta). 20 mL samples were withdraw from the storing glass tank at desired time intervals. After filtration through 0.20 μm membranes, the taken samples underwent several analyses including IC, HPLC, TOC, TN and COD.

## 3. Results and Discussion

Many inorganic species are dissolved in urine, which results in a good ionic conductivity making from EO a suitable method for its treatment (without addition of chemicals). Urine contains generally microorganisms excreted by human body further to its organic and inorganic content. It is important to investigate the electrochemical treatment of synthetic urine wastewaters in presence of *E. coli* as pathogenic microorganisms targeting the transformation of organic content into less harmful compounds and the disinfection of the treated wasters to deactivate the microorganisms. Literature indicated that the nature of the anode material strongly influences the performance of electrochemical oxidation processes [6,55,60] and the nature of disinfection by-products. Different anode materials (Boron doped diamond, iridium oxide, ruthenium oxide and platinum) were utilized to treat synthetic urine wastewaters in presence of *E. coli*.

**Figure 1:**
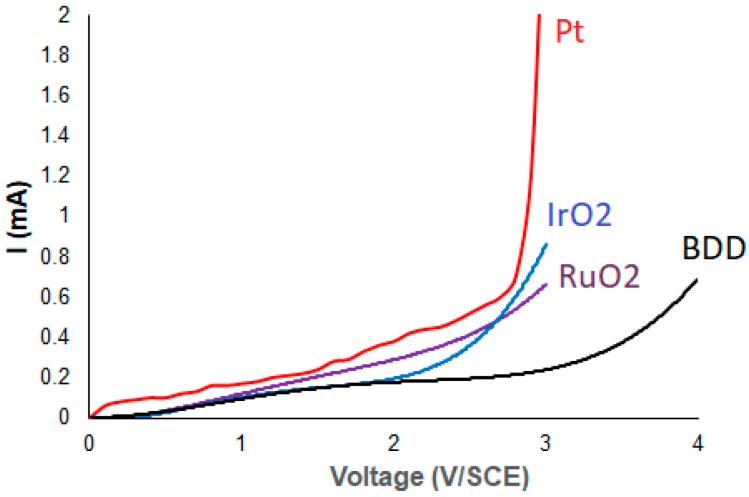
Linear sweep voltammetry of urine synthetic wastewaters using different anode materials. Reference Electrode: SCE, Counter electrode: Graphite, Scan rate: 50 mV/s, Temperature: 25 °C.

Figure 1 presents the linear sweep voltammograms of urine synthetic wastewaters at different anode materials. As it can be seen at Figure 1, the studied electrodes were characterized by different potential of oxygen evolution. BDD is characterized by much higher overpotential oxygen evolution than Pt, RuO_2_ and IrO_2_ electrodes. No anodic peaks were observed for all anode materials indicating that no observable direct oxidation of urine content can occur. 

Similar electrochemical behaviour was observed for all anode materials. The electrochemical activity was analysed at two potential windows: i) one where no oxygen evolution takes place, so organic matter is oxidized directly, based on electron transfer between the electrode surface and the adsorbed substrate and ii) another where high current densities are employed to generate oxygen entities for the mediated oxidation of organic pollutants. As expected, the results clearly indicate that urine components could only be indirectly oxidized on these anodes at the oxygen evolution potential. 

**Figure 2:**
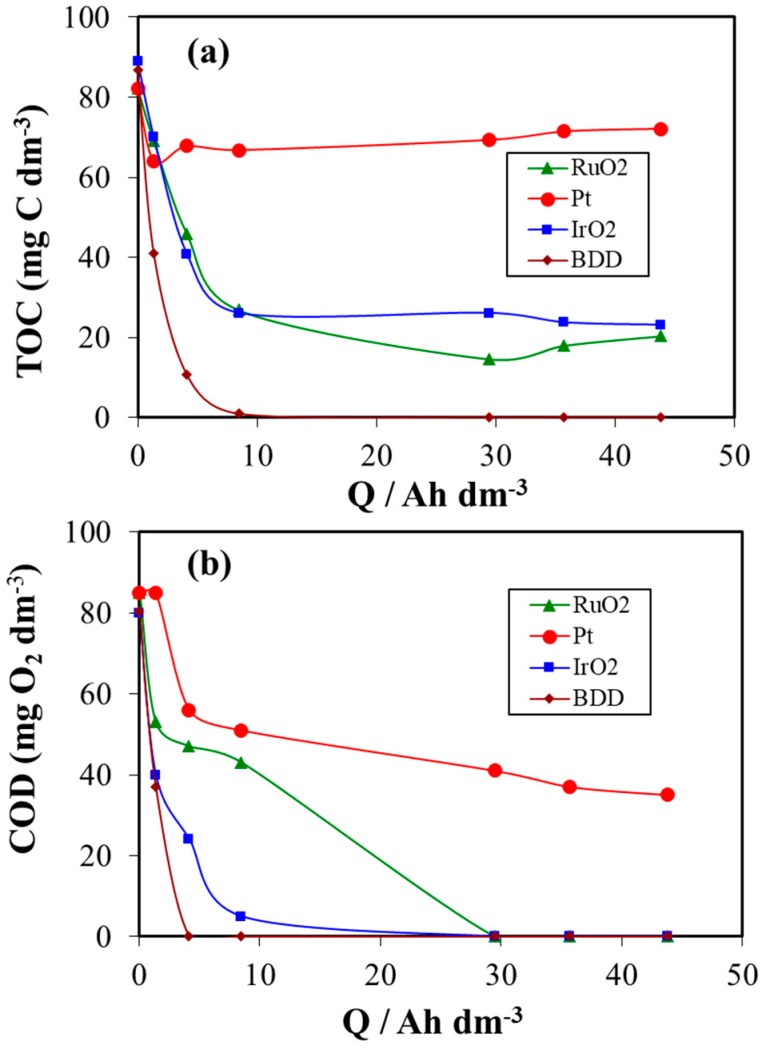
Oxidation and mineralization progress during the electrochemical treatment of urine wastewater at different electrodes.

Figure 2 shows the changes in the concentration of TOC (part a) and COD (part b) with the electric charge applied (Q in Ah dm^−3^) during the electrochemical treatment of urine wastewaters at a constant current density of 15 mA cm^−2^, using different anode materials under similar operating conditions (pH, temperature, organic and inorganic load). Some important differences can be noticed between the performances of four anode materials during the treatment of urine wastewaters. As it can be observed, the use of platinum anode leads to a slow decay of TOC and COD, reaching a poor mineralization and degradation yields of 13 % and 58 %, respectively after 29 h of treatment time (Q ≈ 44Ah dm^−3^). In contrast, both parameters (TOC and COD) decrease rapidly with the applied electric charge during the electrochemical treatment for BDD, IrO_2_ and RuO_2_ anodes. The decay of TOC during the electrochemical treatment indicates the conversion of organic carbon into carbon dioxide CO_2_, while the reduction of COD concentration corresponds to the degradation of the initial products and the formation of intermediates chemical species during the treatment. However, high COD and TOC removal have been obtained by BDD anode. For instance, the TOC removal percentages were realized to be approximately 73 % for the IrO_2_ anode and 82 % for the RuO_2_ anode after consumption of 29 Ah dm^−3^ (24 h). Another important observation is that total removal of TOC was achieved only at BDD anode after consumption of 8 Ah dm^−3^. In addition, total removal of COD was achieved with DSA (IrO_2_ and RuO_2_) anodes after consumption of 29Ah dm^−3^, while 4 Ah dm^−3^ (3 h of electrolysis) were consumed with BDD anode to completely deplete COD. These results clearly show higher performance of BDD electrode for the removal of this complex pollutant than IrO_2_, RuO_2_ and Pt electrodes. This confirms the important role of hydroxyl radicals generated on the surface of BDD anode materials in the overall electrochemical process. As reported in previous research [17,24,25,45,61], BDD anode is characterized by weaker adsorption properties, much higher oxygen evolution potential and a great capability to produce highly reactive radical species from the electrochemical oxidation of water compared to IrO_2_, RuO_2_ and Pt electrodes. Several researchers indicated that BDD anodes can be successfully used to treat wastewater contaminated with organic pollutants [14,17,61,62,63,64]. The comparison with the different material anodes shows that the BDD anode was most efficient for this treatment by electrooxidation/electrodisinfection processes than Pt, IrO_2_, RuO_2_ anodes due to larger electrogeneration of highly reactive hydroxyl radicals, HO^•^. 

**Figure 3:**
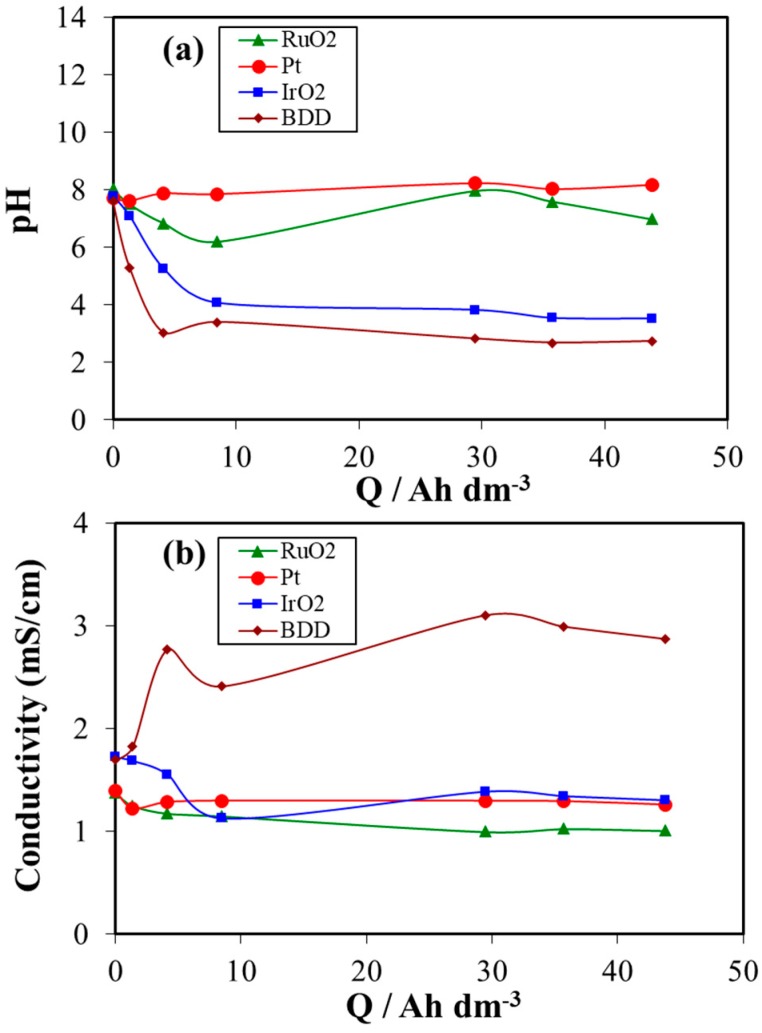
Changes in the (**a**) pH and (**b**) conductivity of the electrolyte with the applied electric charge during the electrochemical treatment of urine wastewaters at four different anode materials.

Figure 3 presents the changes of pH and conductivity of the electrolyte with specific electric charge during electrooxidation/electrodisinfection treatment of urine wastewaters at the four different anodes used in this work. This figure shows that the conductivity of the treated urine did not significantly change during the electrolyses using Pt, IrO_2_, RuO_2_ as anode materials. This result indicates no formation of additional ionic species during the electrochemical treatment of urine wastewaters by Pt, IrO_2_, RuO_2_ anodes. By contrast, the conductivity has increased from 1.7 to 3.1 mS/cm when BDD anode was used. This may be due to the release of inorganic ions from the mineralization of organic nitrogen or from the oxidation of salts present in urine wastewaters during the electrochemical treatment. Additionally, a significant difference in the pH changes was observed between the four anode materials used as shown in Figure 3a. The pH was maintained constant around 8 (initial pH value of urine wastewaters) during the electrochemical process with Pt electrode. In the case of RuO_2_ anode, the pH has a little decrease from pH ≈ 8.0 to pH ≈ 6.2 after 8 Ah dm^−3^ during the experiment. Furthermore, for both electrodes BDD and IrO_2_, the pH decreased rapidly from pH ≈ 8.0 to pH ≈ 3–4 when the electrolysis started and then it remained unchanged until the end of treatment. The decrease of pH during the treatment of urine wastewaters by electrooxidation/ electrodisinfection is related to the electrochemical reactions taking place on the surface of the anode (Equations (2) and (3)) [49,65,66].

H_2_O → HO^•^ + H^+^ + 1e (2)

2H_2_O → O_2_ + 4H^+^ + 4e (3)

This acidic pH is expected to have an important role in the speciation of the intermediates and, as a key point, it helps to prevent the volatilization of ammonia during the electrolysis. Thus, it can be concluded that the change of pH depends largely on the type of electrode material. 

**Figure 4:**
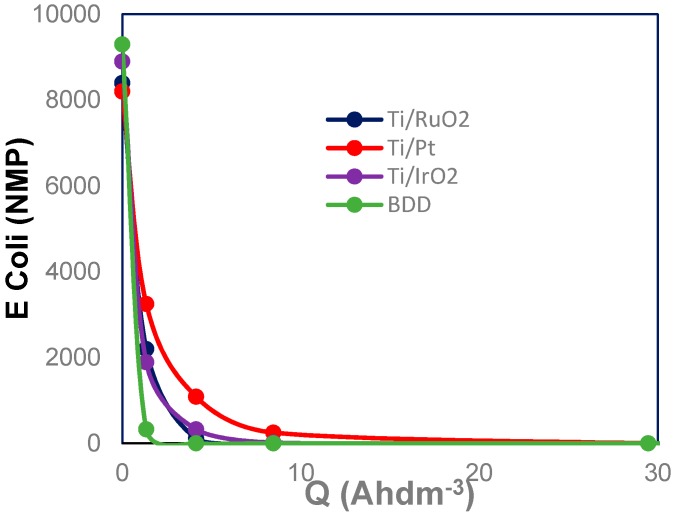
Variation of the *E. coli* concentration with the applied electric charge during the electrochemical treatment of urine wastewaters at four different anode materials (j = 15 mA cm^−2^). *E. coli*: 8300–8700 MNP 100 mL^−1^, T: 25 °C, pH ≈ 8.00.

Figure 4 presents the changes in concentration of *E. coli* with the applied electric charge during electrooxidation and electrodisinfection process of urine wastewaters using four different anode materials (Boron doped diamond, iridium oxide, ruthenium oxide and platinum), at a fixed current density of 15 mA cm^−2^. 

This figure shows that no significant differences in the disinfection performance, regardless of the anode materials used. As it can be seen, the concentration of *E. coli* decreases rapidly at the beginning of the experiments with the applied electric charge (Q). However, the integrated of electrodisinfection and electrooxidation processes with different anode materials (BDD, IrO_2_, RuO_2_ and Pt) allows the complete elimination of the microorganisms in the urine wastewaters at low applied electric charges (1.34 Ah dm^−3^). The total removal of microorganisms in wastewater seems to be directly related with the higher production of disinfectants during electrolysis. These produced disinfectants depend on the concentration of chlorides and the concentration of reduced nitrogen species contained in the urine wastewaters. Furthermore, the electrochemical treatment transforms the organic matter into carbon dioxide, water and inorganic ions. The most important ions that are present in urine treated wastewaters are sulphates, phosphates, nitrates, ammonium and chlorides. 

**Figure 5:**
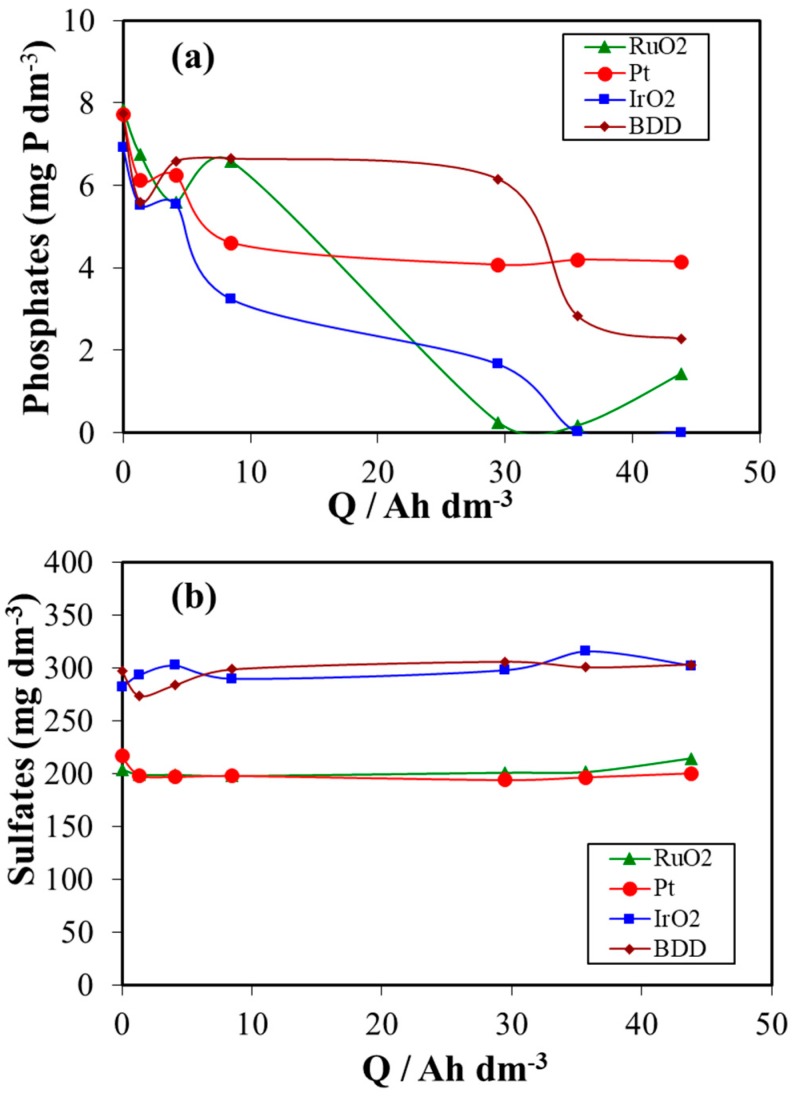
Changes in the concentration of (**a**) phosphates and (**b**) sulphates with the applied electric charge during the electrochemical treatment of urine wastewaters at different anode materials.

Figure 5 shows the changes in the concentration of phosphate (a) and sulphate (b) with the electric charge applied (Q in Ah dm^−3^) during the electrooxidation and electrodisinfection of urine wastewaters at different electrode materials. As seen, phosphate concentration decreased with the applied electric charge for three electrode materials (BDD, IrO_2_ and RuO_2_), while it did not change too much for platinum anode. The decrease in the concentration of phosphates can be correlated with acid-base distribution and solubility of phosphates (PO_4_^3−^, H_2_PO_4_^−^, HPO_4_^2−^, H_3_PO_4_) with pH change and to the formation of perphosphates [67,68,69]. In contrast, sulphates concentration did not change during electrolysis of urine wastewaters for all anode materials used. 

**Figure 6:**
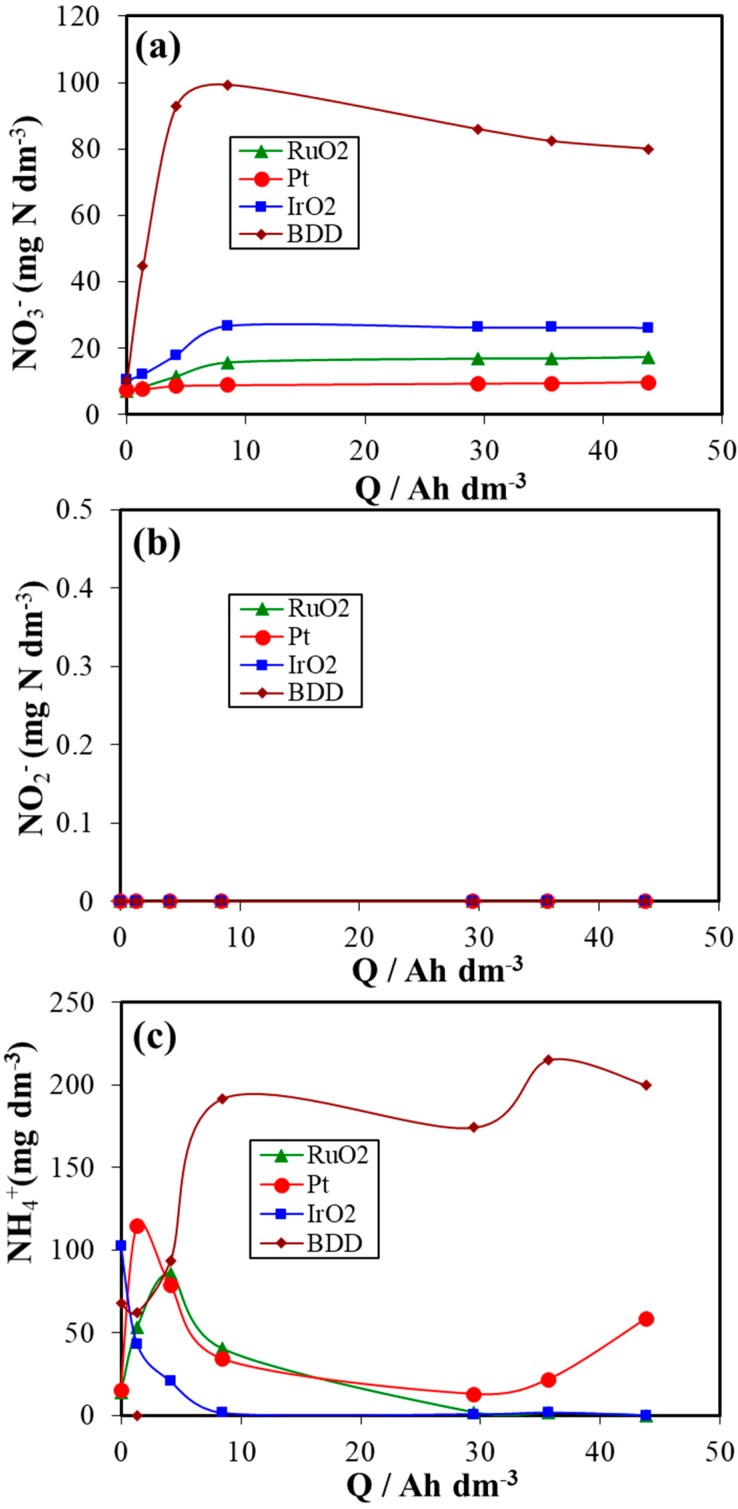
Speciation of nitrogen species during the electrochemical treatment of urine wastewaters with different anode materials. (**a**) Nitrates, (**b**) Nitrites, and (**c**) Ammonium.

Figure 6 shows the evolution of the concentration of nitrogen speciation (nitrates, nitrites and ammonium) with the applied electric charge during the treatment of urine wastewaters at different electrodes (BDD, IrO_2_, RuO_2_ and Pt). As it can be observed, nitrate and ammonium are produced from the beginning of treatment for all anode materials used. In contrary, no nitrite formation was observed during the treatment. 

In this treatment, the initial concentration of nitrogen in urine wastewaters is about 489 mg dm^−3^ and this nitrogen is mostly associated with urea in raw synthetic urine (>95%) being the rest free ammonium ion (6 mg dm^−3^) and organic nitrogen (associated with creatinine and uric acid). However, there is an important differences observed between the BDD anode and the other three anodes (IrO_2_, RuO_2_ and Pt) in terms of the concentration of nitrate and ammonium. It should be noted that the concentration of nitrate attains about 100 mgN dm^−3^, 27 mgN dm^−3^, 16 mgN dm^−3^ and 9 mgN dm^−3^ and the concentration of ammonium reaches about 168 mgN /L, 34 mgN /L, 67 mgN /L and 89 mgN /L for BDD, IrO_2_, RuO_2_ and Pt anodes, respectively. Different performance was achieved depending on the anode material. The concentration of nitrates was higher than the maximum contaminant level of 10 mg dm^−3^ established by environmental agencies for DSA and BDD anodes. This indicates that a post-treatment is needed to decrease the concentration of nitrates. Another option is to replace the stainless steel by carbon-based or Cu/Zn alloy materials [70,71]. The highest concentrations of inorganic nitrogen was measured in the case of BDD anode, though the total conversion of organic nitrogen into organic was not achieved. This might be due to the formation of volatile nitrogen compounds such as nitrogen gas N_2_, ammonia NH_3_ or nitrogen oxides NO_x_ that escape from the reactor [70]. In addition, the rapid reduction of nitrates into ammonium ions and the quick oxidation of chlorides into hypochlorites during the electrolyses are reported by several authors [26,32,41,43,45,55]. The chemical reaction of ammonium ions with hypochlorite ions involves the formation of chloramines and nitrogen gas in the solution according to Equations (4)–(11) [24,25,26,43,72].

NH_4_^+^ + 3HClO → N_2_ + 3H_2_O + 5H^+^ + 3Cl^−^ (4)

NH_4_^+^ + OH^−^ + HClO → NH_2_Cl + 2 H_2_O (5)

NH_2_Cl + HClO → NHCl_2_ + H_2_O (6)

NHCl_2_ + HClO → NCl_3_ + H_2_O (7)

4 NH_2_Cl + 3 Cl_2_ + H_2_O → N_2_ + N_2_O + 10 HCl (8)

NH_2_Cl + NHCl_2_ → N_2_ + 3 HCl (9)

NH_2_Cl + NHCl_2_ + HOCl → N_2_O + 4 HCl (10)

2 NH_2_Cl + HOCl → N_2_O + 3 HCl (11)

**Figure 7:**
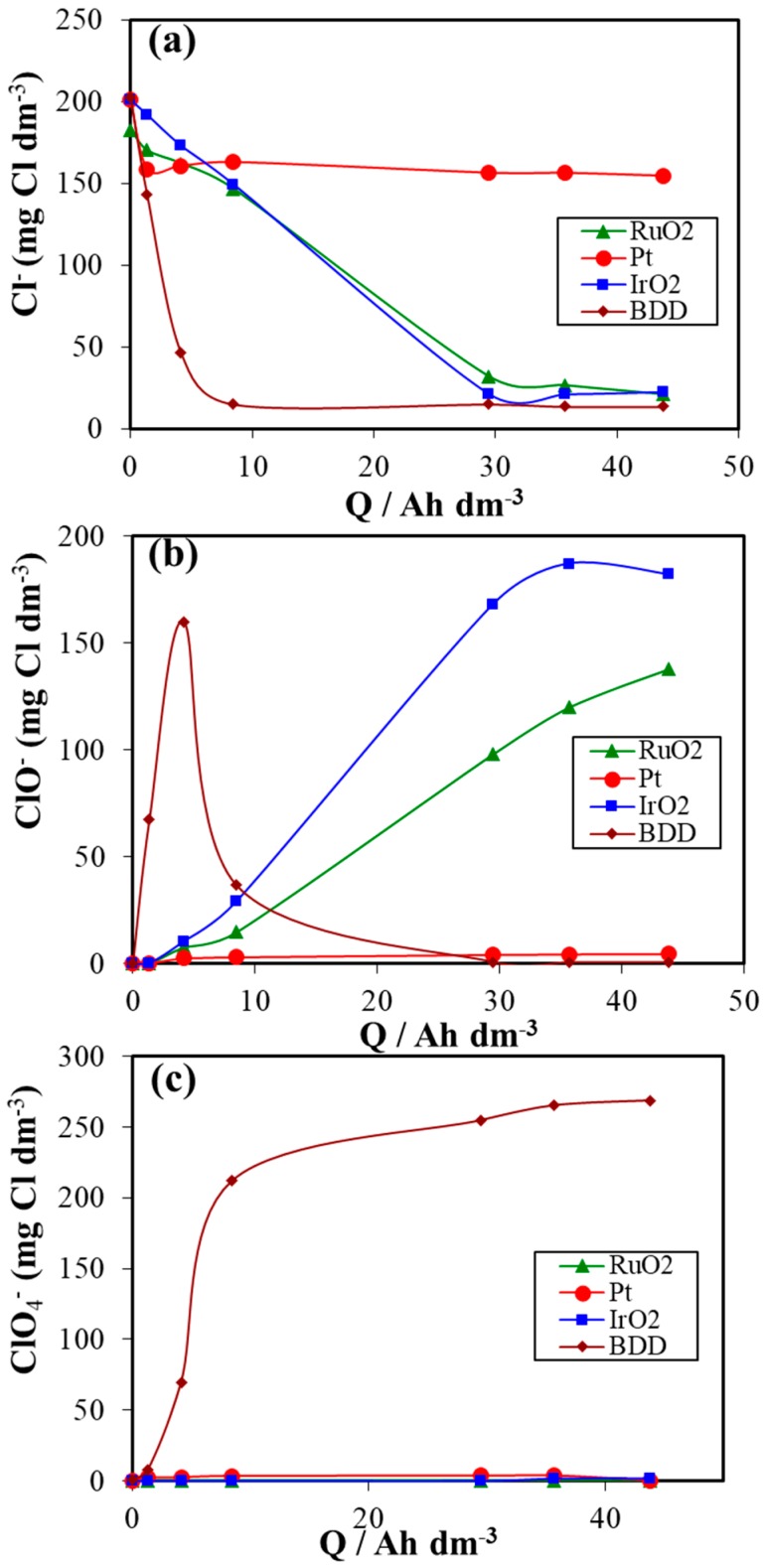
Speciation of chlorine during the electrochemical treatment of urine wastewaters with different anode materials. (**a**) Chlorides, (**b**) Hypochlorites, (**c**) Perchlorates.

Figure 7 shows the speciation of chlorine during the electrochemical process with BDD, IrO_2_, RuO_2_ and Pt anodes materials. As it can be seen from this figure, very important differences are observed between the four different electrodes studied in terms of speciation of chlorine. Oxidation of chlorides is very effective in the case of BDD anodes. It can be also observed that the concentration of chloride is almost constant during the electrodisinfection process of urine wastewaters for the platinum anode. In contrast, during the electrooxidation and electrodisinfection processes with BDD, IrO_2_ and RuO_2_ anode materials, chloride concentration decreased rapidly during the very first moments of the treatment. Removal of chloride is almost complete at 8 Ah dm^−3^ for BDD anode and 29Ah dm^−3^ for IrO_2_ and RuO_2_ anodes. This decrease in the concentration of chloride is due to elimination of microorganisms and its oxidation to produce species in higher oxidation state (hypochlorite, chlorate, perchlorate and other volatile chlorine derivatives) according to Equations (12)–(15) [72,73,74,75,76,77].

Cl^−^ + H_2_O → HClO + H^+^ + 2e^−^ (12)

HClO^•^ → ClO^−^ + H^+^ (13)

2HClO + ClO^−^ → ClO_3_^−^ + 2H^+^ + 2Cl^−^ (14)

ClO_3_^−^ + OH^•^ → ClO_4_^−^ + H^+^ + e^−^ (15)

The concentrations of these species (chlorides, hypochlorite and perchlorates) are presented in Figure 7b,c. Hypochlorite plays a key role in the disinfection process, which is carried out in the electrochemical cell. It facilitates the mineralization of synthetic urine, the elimination of microorganisms and the chlorination of ammonia yielding chloramines until reaching the chlorine breaking point and the removal of the associated nitrogen in the form of nitrogen gas during the electrochemical process. The formation of hypochlorite ions is mainly related to the simultaneous oxidation by hydroxyl radicals and electrooxidation of chloride ions using BDD, RuO_2_ and IrO_2_ anodes [22,23,55,78,79]. As it can be observed, the use of BDD, IrO_2_ and RuO_2_ anodes leads to the generation of hypochlorite during the electrodisinfection process but only small amounts of hypochlorites (do not exceed 7 mg Cl dm^−3^) were observed during the treatment for Pt anode. Also, for BDD anode, the concentration of hypochlorites increases rapidly at the beginning of the treatment, to reach a maximum of about 160 mg Cl dm^−3^ at 4 Ah dm^−3^ and then drops sharply and completely disappears after 8 Ah dm^−3^. This decrease indicates the attack to *E. coli* (causing their death), the oxidation of hypochlorites by hydroxyl radicals to from chlorates and then perchlorates according to Equations (12)–(15). It should be noted that perchlorates can pose an environmental threat due to their high toxicity if they are not well-controlled [80,81]. Regarding the production of perchlorates, no formation of perchlorates was observed for the three different anode materials (Pt, IrO_2_ and RuO_2_). 

**Figure 8:**
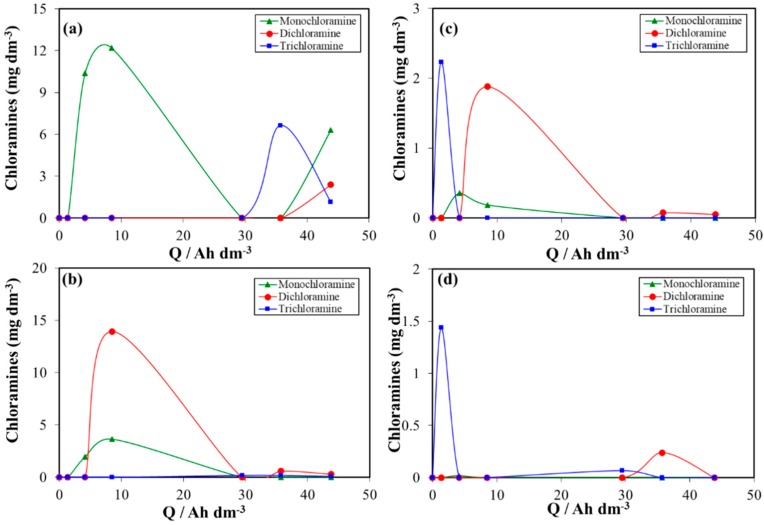
Changes in the concentration of chloramines (▲ monochloramine; ● dichloramine; ■ trichloramine) with the applied electric charge during the electrochemical treatment of urine wastewaters with (**a**) RuO_2_, (**b**) IrO_2_, (**c**) Pt and (**d**) Boron doped diamond (BDD) anode materials.

Figure 8 presents the changes in the concentration of chloramines with the applied electric charge during electrolyses of urine wastewaters at different electrode materials. 

As can be observed, the concentration of chloramines measured in all experiments was very low and did not significantly increase with the applied electric charge during the electrochemical treatment using BDD, IrO_2_, RuO_2_ and Pt electrodes. The low formation of chloramines is mainly due to acidic pH conditions [32,33,75]. Chloramines can play an important role in shifting reactions (8)–(11) towards the formation of nitrogen gas and in deactivating microorganisms for longer lasting time than chlorine. Hence, the electrochemical technology based on BDD and DSA anodes can be utilized not only for the removal of organic pollutant but also for the removal of nitrogen from urine wastewaters. 

## 4. Conclusions

The electrochemical treatment of synthetic urine wastewaters with boron-doped diamond (BDD), dimensionally stable anode (DSA: IrO_2_ and RuO_2_) and platinum (Pt) electrode materials allows to remove completely the *E. coli* present in urine wastewaters at low applied electric charge (1.34 Ah dm^−3^). Complete depletion of COD and TOC can be obtained during electrolyses with BDD anode at applied electric charge of 4 Ah dm^−3^ and 8 Ah dm^−3^, respectively. On the other hand, with DSA anodes (IrO_2_ and RuO_2_), the removal of TOC and COD was achieved but after consumption of high electric charge, while for Pt anode, low TOC and COD were eliminated during electrochemical oxidation/disinfection. The highest production of ammonium and nitrates was observed during the electrochemical treatment of urine wastewaters using BDD anode. BDD and DSA anodes oxidize the chlorides present in urine wastewaters into hypochlorite capable to kill the microorganisms. Large amounts of chlorate (ClO_3_^−^) were measured during electrolysis using DSA anodes. Perchlorate (ClO_4_^−^) ions were detected only when BDD was used as anode materials. Low concentration of chloramines was monitored in all experiments. The electrooxidation using BDD and DSA anodes is an efficient technology to eliminate organic content, to deactivate *E. coli* microorganisms in urine wastewaters. It can also transform organic nitrogen efficiently into inorganic ions and oxidize chlorides in hypochlorites. However, high amounts of chlorates and perchlorates were measured at the end of electrolysis. The combination of electrooxidation using BDD and DSA anodes with UV photolysis or sonication can avoid the formation of chlorates and perchlorates if experimental conditions are optimized.
